# Influence of Pulsed Electric Field on Accumulation of Calcium in *Lactobacillus rhamnosus* B 442

**DOI:** 10.4014/jmb.1908.08064

**Published:** 2019-12-09

**Authors:** Maógorzata Głral, Urszula Pankiewicz, Monika Sujka, Radosław Kowalski, Dariusz Góral, Katarzyna Kozłowicz

**Affiliations:** 1 Department of Analysis and Food Quality Assessment, Faculty of Food Science and Biotechnology University of Life Sciences, Skromna 8, 20-704, Lublin, Poland; 2 Department of Biological Bases of Food and Feed Technologies, Faculty of Production Engineering, University of Life Sciences, Głęboka 8, 0-61, Lublin, Poland

**Keywords:** PEF, calcium, *Lactobacillus rhamnosus*

## Abstract

Calcium is an element that performs many important functions in the human body. A study was conducted on the use of a pulsed electric field (PEF) to enrich cells of *Lactobacillus rhamnosus* B 442 in calcium ions. The highest concentration of calcium ions in bacterial cells (7.30 mg/g d.m.) was obtained at ion concentration of 200 μg/ml of medium and with the use of the following PEF parameters: field strength 3.0 kV/cm, exposure time 10 min, pulse width 75 ms and 20 h of culturing after which bacteria were treated with the field. Cell biomass varied in the range from 0.09 g/g d.m. to 0.252 g/g d.m., and the total number of bacteria ranged from 10^10^ CFU/ml to 10^12^ CFU/ml. Microscope photographs prove that calcium ions were situated within the cells of the bacteria, and electroporation contributed to an increase in the effectiveness of the ion bioaccumulation process. Samples containing calcium and subjected to electroporation displayed intensive fluorescence. The significance of this research was the possibility of using probiotic bacteria enriched with calcium ions for the production of functional food in subsequent studies.

## Introduction

Calcium participates in many physiological processes, *e.g.* in the contraction of skeletal, smooth and heart muscles, and the conduction of nerve impulses and blood clotting [1]. Osteoporosis, caused by the lack of calcium absorption irrespective of age, afflicts 75 million people worldwide [2].

There are many factors that affect calcium assimilability from food products. Oxalates and phytates present in food products of plant origin reduce its bioavailability, while sugars and proteins increase the absorption of Ca^2+^ ions. A high content of sodium in the diet increases urinary excretion of calcium [3]. Calcium is absorbed mainly in the intestines and, to a small extent, in the stomach, and comprises two mechanisms: passive diffusion and active transport. Passive diffusion depends on the gradient of concentration of free calcium cations. Active transport, on the other hand, requires the presence of vitamin D, transport proteins and energy [1, 4]. Pharmacological supplementation of the diet with calcium ions increases the risk of developing renal calculosis, cardio-vascular diseases and damage to the kidneys. Diet supplements containing calcium may enter into interactions with medicines or cause disturbance to the alimentary tract [5-7]. Bacterial cell biomass enriched with calcium has become an alternative for pharmacological supplementation applied in the deficit of those cations. Bioelements administered in the form of metalloproteins are more easily assimilable by the organism compared to pharmaceutical preparations. Food products produced with the participation of bacteria enriched with calcium could provide, in the future, an additional source of these in the diet [8].

Until recently, PEF technology was used solely for the stabilization of food products [9, 10]. Lately, however, more and more reports are being published concerning research on the use of PEFs to generate specific effects in biological systems [11]. A pulsed electric field has a direct impact on the permeability of cellular membranes. Suitably optimized parameters for PEFs (electric field strength, pulse duration, number of pulses applied and frequency)[12] may produce therapeutic effects through the stimulation of temporary release of medicines, enhanced healing of wounds, and restoration of tissue integrity [13]. Pulse duration usually varies from nanoseconds to a millisecond, and field strength – from several kV/cm to hundreds or thousands of V/cm [14, 15]. Apart from the field parameters, the permeability of cellular membranes is affected also by the composition of the buffer used for the electroporation, temperature, and cell properties (size, shape, adherence and density). In the process of reversible electroporation, a PEF induces transitional permeability of the cellular membrane, as a result of which permanent structures are formed (often referred to as “pores” or “nanopores”) which facilitate the exchange of components with the environment of the cell. It is then possible to introduce into the cytoplasm of cells various chemicals, hydrophilic drugs, or large and charged molecules, such as DNA. The increased permeability of the cellular membrane can last from several seconds to even several hours after the application of a PEF [16]. Pulsed electric fields can have an impact on intracellular structures. Suitably selected parameters increase gene expression, induce DNA damage, and cause calcium liberation from cellular organelles [17]. Potentially, a nanosecond pulsed electric field can also stimulate the voltage-gated ion (calcium) channels (VGCC), which are the main entry pathways of Ca^2+^ into the cell [18]. However, irreversible electroporation causes permanent disintegration of cell membrane, leading to the death of the cell [19].

The objective of the study presented here was to identify the optimum parameters of pulsed electric fields to increase the intracellular calcium content while preserving a high survival rate for cells of *Lactobacillus rhamnosus* B 442.

## Materials and methods

### Materials

A bacterial strain of *Lactobacillus rhamnosus* B 442 from the Agricultural Research Service Culture Collection NRRL (WDCM97) maintained at the Department of Biotechnology, Human Nutrition and Science of Food Commodities, University of Life Sciences and Biotechnology in Lublin, Poland, was used in the experiment. For the preparation of inoculum and culture medium the following components were used: sterile MRS broth (Biocorp, Warszawa, Poland) 59.937 g/l, agar (DIFCO, USA) 15 g/l, NaCl 80 g/l, glycerol (TechlandLab, Tarnobrzeg, Poland), HNO_3_ 65% (Merck, Germany) and CaCl_2_ (Standard, Poland), in fixed concentrations.

### Cultivation of Bacteria

Bacteria were passaged three times in MRS broth and incubated for 19 h at 37°C. Then 25 ml of bacteria was transferred to 475 ml of sterile medium in 1,000-ml Erlenmeyer flasks and incubated at 37°C for 16 h.

### Optimization of Calcium Concentration in Bacteria

Optimization of calcium concentration in the medium was performed by culturing *L. rhamnosus* B 442 at different concentrations of calcium (μg/ml medium): 10, 25, 100, 150, 200, 400, 500, 750, 1,000. For this purpose, the inoculum was incubated for 48 h. Then it was centrifuged and the medium was removed. In order to obtain the abovementioned concentrations of calcium in the medium, 10 ml of the ion solution at the appropriate concentration was added to 90 ml of deionized water containing the inoculum. The cultures were treated with PEFs for 15 min at pulse width 20 μs, electric field strength 2.0 kV/cm, using a laboratory electroporator (BTX Harward Apparatus, model ECM 830). Simultaneously the control cultures were conducted, respectively, K1 – without calcium added to the medium and without PEF treatment, and K2 – with calcium added to the medium in the concentrations mentioned above and without PEF. The PEF treatment chamber consisted of four parallel plexiglass plates that had stainless steel electrodes with an area equal to 4 cm^2^, facing each other with a gap of 5 mm. The culture was agitated in the chamber during PEF treatment by means of a magnetic stirrer.

### Optimization of PEF Parameters

Optimization of the electric field parameters was carried out in several stages. In the first stage, the strength of the electric field was optimized by subjecting the culture, after 16 h of incubation, to the action of a field strength in the range of values from 0.1 to 1.0 kV/cm and from 1.5 to 6.0 kV/cm, exposure time of 15 min, pulse width of 20 μs and frequency of 1 Hz, at calcium concentration of 200 μg/ml of medium, which was set earlier as described in the section above (Optimization of Calcium Concentration in Bacteria). Then the time of exposure to the PEF was optimized in the range of 5-25 min, at optimal electric field strength (3.0 kV/cm). In the following step, pulse width was optimized in the range of 10 150 μs at optimal PEF parameters: electric field strength 3.0 kV/cm, electroporation time 10 min and frequency 1 Hz. In the last stage, optimization of the time of incubation after which bacterial cells were treated with the PEF was performed. Cells were electroporated after 8, 12, 16, 20, and 24 h of culturing. One of the samples was subjected to a multiple treatment with PEF after 8, 12, 16, 20, and 24 h of culturing. In both cases the optimal PEF parameters were applied: electric field strength 3.0 kV/cm, time of exposure to PEF 10 min, pulse width 75 μs, and frequency 1 Hz.

### Determination of Calcium Concentration

Determination of calcium concentration in bacterial biomass after mineralization was performed by atomic absorption spectro-photometry (ET-AAS, VARIAN AA 280 FS, Agilent, USA) according to the procedure described by Jorhem and Engman [20] and Góral *et al*. [23].

### Visualization of Calcium Ions in Cells

Visualization of calcium ions in bacterial cells was performed by confocal microscope (Nikon Eclipse, Netherlands). Pigment solution was prepared in accordance with the recommendations of the manufacturer. Two millimolars of calcium orange (Invitrogen, USA) was dissolved in anhydrous DMSO. A small amount of bacteria (approx. 0.5 ml) was placed in an Eppendorf tube filled with 0.5 ml of PBS and then the microorganisms were dissolved in the solution by shaking. Twenty microliters of the prepared calcium orange solution was added to the bacterial suspension. The specimen for microscope observation was prepared by spreading the dyed bacterial suspension on a cover slide (without covering) and drying. An argon laser with a wavelength of 488 nm was used to induce fluorescence.

### Determination of Total Number of Microorganisms

Total number of microorganisms was determined by plate dilution method according to American Public Health Association [21] and Góral and Pankiewicz [22].

### Determination of Biomass

Biomass was determined spectrophotometrically (Spekol 11, Carl Zeiss, Germany) according to the procedure described by Góral *et al*. [23].

### Statistical Analysis

All determinations were made in triplicate. Significant differences between individual groups were found using the Student’s *t*-test at the level of significance α = 0.05. Statistical processing of results was carried out using the R version 3.1.2 (GNU General Public License, USA).

## Results 

### Concentration of Ca^2+^ Ions and Increase of Biomass at Variable Concentrations and Optimized PEF Parameters

The bacterial cell is a natural absorbent of metal ions due to the unique composition of the cellular membrane. Ions are bound thanks to two mechanisms: biosorption and bioaccumulation. This paper presents the results concerning both surface binding of calcium ions and their incorporation in cellular structures.

**Fig. 1:**
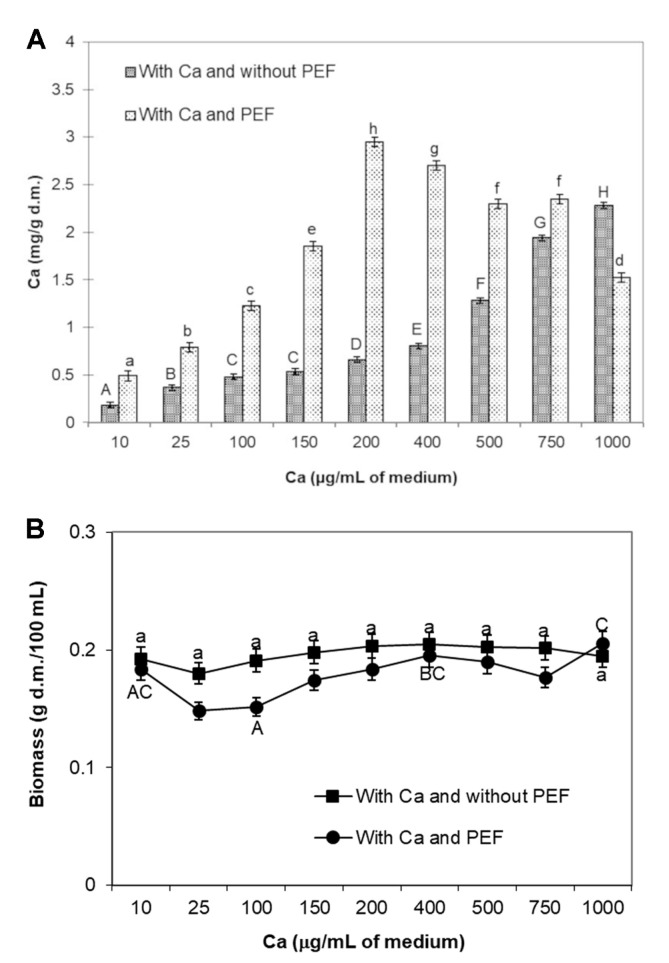
Effect of optimization of Ca^2+^ ion concentration the following: (**A**) Calcium concentration in cells of *L. rhamnosus* B 442. (**B**) Biomass content in cultures of *L. rhamnosus* B 442. Applied process parameters: electric field strength 2.0 kV/cm, pulse width 20 μs, field frequency of 1 Hz, the time of exposure to PEF 15 min after 20 h culturing. Means with the same lower case or capital letters are not highly significantly different (*p* < 0.05; *n* = 9).

Within the entire range of applied calcium concentrations (10-1,000 µg/ml of medium), the concentration of ions in cells not subjected to the effect of a PEF increased with increase of the concentration of Ca^2+^ in the medium and attained from 0.183 mg/g d.m. (at 10 µg/ml of medium) to 2.28 mg/g d.m. (1,000 µg/ml of medium). For comparison, in the control sample K1 (with no addition of calcium and PEF application) the content of the ions was 0.127 mg/g d.m. The application of pulsed electric field caused, within the concentration range of 10 to 750 µg/ml of medium, an increase in the accumulation of calcium ions in the cells (compared to cultures not subjected to PEF): 2-fold (for concentrations of 25 and 100 µg/ml of medium), and even 4-fold (for 200 µg/ml of medium). Only at the concentration of 1,000 µg/ml of medium in cells treated with PEF a lower accumulation of calcium ions was noted, compared to cells with no PEF treatment, which was due to the diffusion of ions from the cell to the medium (Fig. 1). Initially, the accumulation of ions in PEF-treated cells increased with increase in calcium concentration in the medium. Above 200 µg/ml of medium, a gradual decrease of this concentration in cells was noted. The highest accumulation of Ca^2+^ ions in cells was achieved by applying the pulsed electric field at calcium concentration of 200 µg/ml of medium (Fig. 1A). Cell biomass in cultures not subjected to the effect of a PEF did not change significantly at the range of applied concentrations and varied from 0.18 ± 0.003 g d.m./ 100 ml to 0.20 ± 0.008 g d.m./100 ml. By contrast, the application of PEF caused a decrease of biomass within the entire range of applied concentrations, with the exception of the concentration of 1,000 µg/ml of medium, at which the highest biomass was noted, 0.21 ± 0.003 g d.m./100 ml (Fig. 1B).

**Fig. 2:**
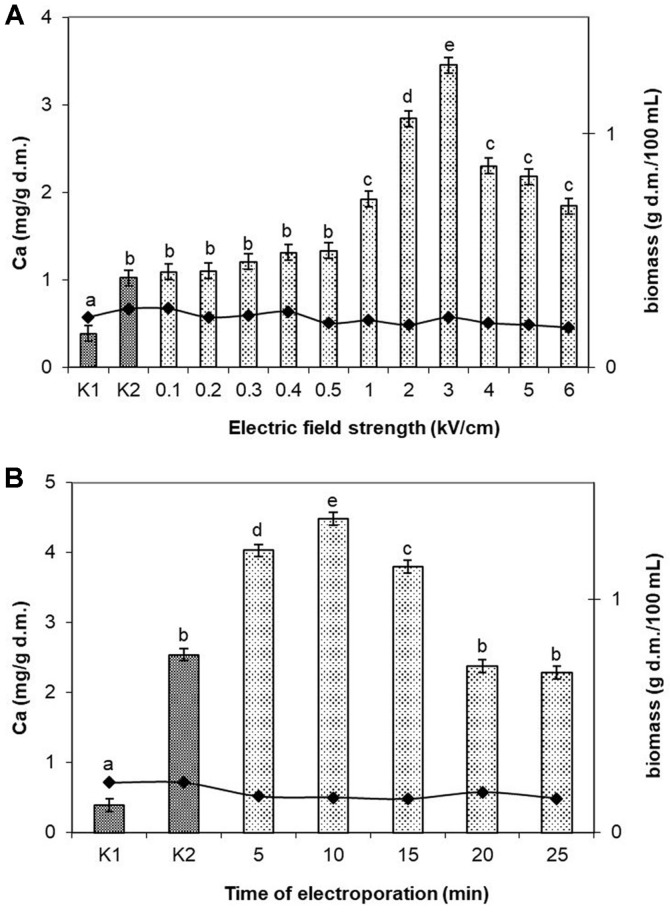
Accumulation of Ca^2+^ in *L. rhamnosus* B 442 in relation to the following: (**A**) Electric field strength (pulse width 20 μs, field frequency of 1 Hz, time of exposure to PEF 15 min after 20-h culturing; calcium concentration in the culture medium 200 μg/ml). Means with the same letters are not highly significantly different (*p* < 0.05; *n* = 13). (B) Time of exposure to PEF (electric field strength 3.0 kV/cm pulse width 20 μs, at field frequency of 1 Hz; after 20- h culturing; calcium concentration in the culture medium 200 μg/ml). Means with the same letters are not highly significantly different (*p* < 0.05; *n* = 7). Control cultures K1: without Ca^2+^ and without PEF treatment; K2: with Ca^2+^ (200 μg/ml) and without PEF

Field strength in the range of 0.1 kV/cm-0.5 kV/cm did not have any statistically significant effect on the accumulation of Ca^2+^ ions, which was comparable to that obtained in the control sample K2 (enriched with calcium ions but without PEF application). A statistically significant increase on ion concentration in cells was observed only at field strength from 1.0 kV/cm to 3.0 kV/cm. The highest concentration of ions in cells (3.45 mg/g d.m.) was achieved by applying field strength of 3.0 kV/cm (Fig. 2A). Further increase of field strength in the range from 4.0 kV/cm to 6.0 kV/cm caused a statistically significant decrease of accumulation, which was due to the diffusion of ions from the cells to the medium. Field strength had no effect on the level of cell biomass which varied from 0.17 g d.m./100 ml to 0.25 g d.m./100 ml, similarly as in the control cultures K1 and K2.

In the next stage, the electroporation time was optimized. Cells were treated with a pulsed electric field for periods from 5 min to 25 min, at the optimum field strength of 3.0 kV/cm (Fig. 2B). Five-minute electroporation caused an increase in the accumulation of ions by over 50% compared to the control culture K2. The highest concentration of ions (4.48 mg/g d.m.) was obtained after treating the culture with a PEF for 10 min. Further extension of exposure time to 15, 20, and 25 min caused a statistically significant decrease in the concentration of ions in cells. The time of exposure did not cause any changes in the bacterial biomass which varied in the range of 0.143 g d.m./100 ml–0.173 g d.m./100 ml, with its mean value being lower by 62% than the biomass in the control cultures K1 and K2.

**Fig. 3:**
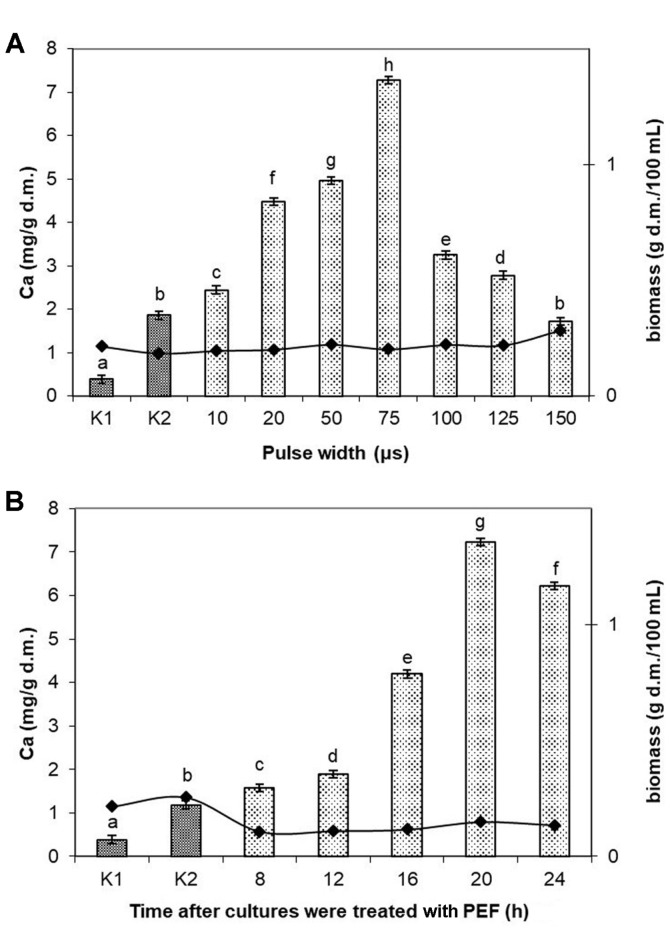
Accumulation of Ca^2+^ in *L. rhamnosus* B 442 cell biomass in relation to the following: (**A**) Pulse width (electric field strength 3.0 kV/cm, at field frequency of 1 Hz, time of exposure to PEF 10 min after 20 h culturing; calcium concentration in the culture medium 200 μg/ml). Means with the same letters are not highly significantly different (*p* < 0.05; *n* = 9). (**B**) Time after cultures were treated PEF (pulse width 75 μs, electric field strength 3.0 kV/cm, at field frequency of 1 Hz, time of exposure to PEF 10 min after 8, 12, 16, 20, or 24 h culturing; calcium concentration in the culture medium 200 μg/ml). Means with the same letters are not highly significantly different (*p* < 0.05; *n* = 7). Control cultures K1: without Ca^2+^ and without PEF treatment; K2: with Ca^2+^ (200 μg/ml) and without PEF.

In the next stage of the study, the pulse width at which the maximum concentration of calcium ions in cells was attained was optimized. Cells were treated with a PEF having a pulse width of 10 µs–150 µs applying the parameters optimized earlier: field strength of 3.0 kV/cm and electroporation time of 10 min. Initially, pulse width increase in the range of 10 µs-75 µs caused an increase in the concentration of ions in the cells. At pulse width of 75 µs the highest accumulation of calcium (7.28 mg/g d.m.) in the cells was obtained, 3.5-fold higher than in the control culture K2, where no PEF treatment was applied. The optimization of the next PEF parameter – pulse width, caused a 63% increase of calcium accumulation in the cells relative to the culture in which the exposure time was optimized (Fig. 3A). No significant differences were shown in biomass content in relation to the pulse width applied. The average content of biomass was 0.21 ± 0.01 g d.m./ 100 ml.

In the final stage, the time after which cells were treated with a pulsed electric field was optimized. Cultures were treated with a PEF after 8, 12, 16, 20 or 24 h from the start of the culturing, using the parameters optimized earlier (field strength of 3.0 kV/cm, exposure time of 10 min and pulse width of 75 µs). The cultures treated with a PEF after 8 and 16 h were characterized by low accumulation, of 1.58 mg/g d.m. and 1.89 mg/g d.m., respectively. The highest concentration of calcium ions in cells (7.30 mg/g d.m.) was obtained after PEF treatment of the 20-h culture (Fig. 3B). PEF treatment of bacteria after 24 h of culturing caused a 17% lower concentration of ions compared with the culture treated with PEF after 20 h, *i.e.* in the phase of logarithmic growth. One of the cultures was subjected to multiple treatments with a PEF (after 8, 12, 16, 20, and 24 h of culturing), attaining accumulation of 2.57 mg/g d.m. Multiple PEF treatments of the culture caused a nearly 3-fold decrease of accumulation compared with culture treated with PEF once at the optimum field parameters. Cell biomass after 8- and 12-h culturing was at a very low level (mean of 0.11 ± 0.001 g d.m./100 ml), which was caused by the short culturing period of the microorganisms. At the optimized time after which cultures were treated with a PEF, the biomass was at the level of 0.14 ± 0.01 g d.m./ 100 ml.

### Estimation of Survival Rate of Bacteria at Variable Ca^2+^ Concentrations and PEF Parameters

Increasing the concentration of Ca^2+^ ions in cells had an impact on the total number of microorganisms only in selected ranges of concentration (Table 1). In the culture supplemented with calcium but not subjected to the effect of a PEF the survival rate of *L. rhamnosus* B 442 did not display any significant differences at concentrations in the ranges of 10 µg/ml-150 µg/ml of medium and 400 µg/ml-1,000 µg/ml of medium, compared to the control culture K1 (without PEF and Ca^2+^). The highest survival rate (1.75·10^12^ CFU/ml) was observed in the culture not subjected to the effect of a PEF and supplemented with calcium at the concentration of 200 µg/ml of medium. In the PEF-treated cultures, the highest total number of microorganisms was noted at the application of concentrations of 25 µg/ml, 150 µg/ml, and 400 µg/ml of medium. The culture in which the highest accumulation of calcium was obtained did not display any significant differences in the survival rate of microorganisms in relation to cultures with concentrations of 10 µg/ml, 100 µg/ml, and from 500 µg/ml to 1,000 µg/ml of medium.

Only in the case of optimization of pulse width did the application of a PEF and the time after cultures were treated with a PEF have an impact on the survival rate of bacteria (Table 2). Both the low and high field strength levels and time of exposure had no statistically significant effect on the total number of microorganisms compared to the control cultures K1 and K2. Pulse widths of 125 µs and 150 µs caused an increase in the survival rate of bacteria, compared to the remaining cultures.

### Visualization of Calcium Ions in Cells

**Fig. 4:**
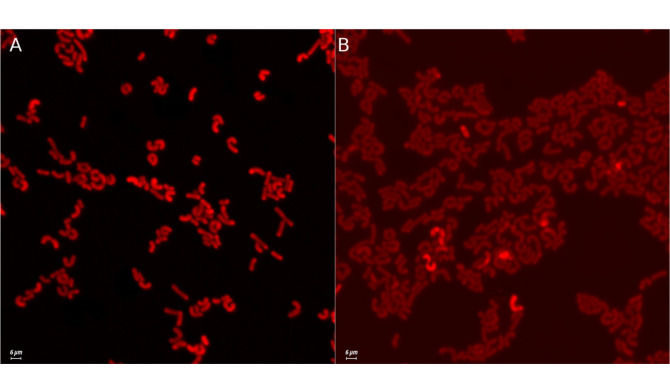
Confocal microscopy images showing fluorescence of calcium in *L. rhamnosus* B 442 cells. (**A**) *L. rhamnosus* B 442 with Ca^2+^ and PEF. (**B**) *L. rhamnosus* B442 without Ca^2+^ (control sample K2).

Microscopy specimens were prepared from cultures subjected to the process of electroporation and from the control cultures K2. Distinct differences were observed among the analyzed specimens (Fig. 4). Specimens containing calcium and subjected to electroporation displayed intensive fluorescence, while in K2 fluorescence was noted only for individual bacterial cells.

## Discussion

Góral and Pankiewicz [22], in bacterial cultures enriched with magnesium ions by means of a PEF, achieved biomass of 0.259-0.315g d.m./100 ml, and in cultures supplemented with zinc ions – from 0.264 to 0.323 g d.m./100 ml (with PEF and Zn^2+^) [23].

Góral and Pankiewicz [22] demonstrated the highest accumulation of magnesium ions (4.28 mg/g d.m.) in cells of the same bacterial strain at magnesium ion concentration of 400 µg/ml of medium, and subjected to the effect of PEF with strength of 2.0 kV/cm, pulse width 20 µs, and electroporation time of 15 min after 20 h of incubation. On the other hand, Góral *et al*. [23], in a study on the concentration of zinc ions in cells of the same strain, noted the highest concentration (2.85 mg Zn/g d.m) at the concentration of 500 µg Zn/ml of medium and application of the optimum PEF parameters: field strength of 3.0 kV/cm, pulse width of 20 µs, electroporation time of 15 min after 20 h of culturing. The accumulation of zinc was lower than that of magnesium or calcium ions, due to the toxic effect of that ion on bacterial cells at high doses. Calcium and magnesium ions support the growth of microorganisms and are a natural admixture to culturing media for lactic acid bacteria [24].

Optimization of pulsed electric field parameters allowed the highest maximum accumulation of calcium ions in cells. In the first stage of the study the field strength was optimized. Field strength largely determines the degree of permeability of the cellular membrane and the reversibility of the process [25]. The pulsed electric field strength used in the above analyses was higher than that used in studies on cells of mammals [26], and lower by more than one half that used in the deactivation of microorganisms. It is assumed that field strength which causes cellular membrane rupturing is at least 15 kV/cm [27]. Pankiewicz and Jamroz [28] noted the highest accumulation of calcium in the yeast *Saccharomyces cerevisiae* at the following parameters: field strength 5 kV/cm, pulse width 20 µs, exposure time 20 min, after 20 h of culturing. Then, concentration of calcium ions in yeast cells was 3 mg/g d.m., *i.e.* 140% less than the level achieved with the use of the optimized PEF parameters in this study. The authors of the available publications conducted research also on the accumulation of other elements in cells of microorganisms. Roman *et al*. [29] demonstrated that an addition of magnesium acetate to culturing medium caused an accumulation of that element in biomass of bacteria *L. brevis* at the level of 2.65–5.82 mg Mg g^-1^s.s and from 1.73 to 3.28 mg Mg g^-1^s.s. relative to *L. plantarum* ATCC 4080. Those results were lower than the accumulation of calcium ions in cells of *L. rhamnosus* B 442. Mörschbächer *et al*. [30], in a study on the estimation of the potential of strains of lactic acid bacteria for bioaccumulation of selenium, observed that *L. paracasei* (ML13 and CH135) bio-accumulated the highest concentrations of Se (38.1 ± 1.7 mg/g and 40.7 ± 1.1 mg/g, respectively) in the presence of 150 mg/l sodium selenite. The bioaccumulation results obtained were notably higher than those presented in the above paper. Mrvčić *et al*. [31], in a study on the capacity for the accumulation of zinc ions by various species of lactic acid bacteria, determined the lowest concentration of that element in cells of bacteria *L. plantarum*. Those authors demonstrated that the accumulation of the ions was related to their concentration in the nutrient medium. The addition of zinc ions at 10 mg/l medium resulted in a higher accumulation of zinc ions in bacterial cells than the addition of zinc in concentration higher than 90 mg/l. With increase in the concentration of the ions (up to 90 mg/l), there was a decrease of their concentration in the biomass, from 70% to 25% for *L. mesenteroides*, from 60% to 14% for *L. brevis*, and from 50% to 13% in relation to *L. plantarum*, respectively. Mrvcic *et al*. [32] noted that the absorption of ions in cells of lactic acid bacteria depended on the concentration of those ions in the solution. Those authors, based on the Langmuir model, observed a high capacity for copper binding by *L. mesenteroides* and *L. brevis*. Similarly to our study, those authors observed that the concentration of ions in the medium has a significant effect on their accumulation.

The obtained survival rate of microorganisms was at a high level, which will allow further storage of bacteria enriched with calcium ions without any loss of their probiotic properties. The definition of probiotics proposed by FAO/WHO (2002) does not specify the minimum amount of probiotics in a product [33]. It is believed, however, that a product can be assumed to be probiotic if it contains at least 10^9^ colony forming units [34].

Ziarno and Wieclawski [35] estimated the effect of calcium salts on the growth of lactic acid fermentation bacteria. They demonstrated that an addition of calcium lactate did not have any significant effect on the growth of lactic acid fermentation bacteria in a culturing medium and in milk. A higher growth dynamic was observed in a culture of lactic streptococci from the inoculum FL-DAN containing *Lactococcus lactis* subsp. *cremoris*, *Lc. lactis* subsp. lactis, *Leuconostoc mesenteroides* subsp. *cremoris*, *Lc. lactis* subsp. *Diacetilactis*, and lactic bacilli from a culture containing *Lc. lactis* subsp. *cremoris*, *Lc. lactis* subsp. *lactis*, *Lc. lactis* subsp. *diacetilactis*, *Leu. mesenteroides* subsp. *cremoris*. The growth of lactic bacilli from the inoculum FL-DAN incubated in milk was significantly inhibited. Pirkul *et al*.[36] demonstrated that an addition of CaL and CaG had a beneficial effect on the survival rate of lactic bacilli in yoghurt. Seratlić *et al*. [37], in a study on the effect of pulsed electric fields on the survival rate of the population of *L. plantarum* 564 observed a significant decrease of the survival rate of the microorganisms at the application of a PEF with the following parameters: 31.6 kV/cm; 5 µs×10 i 31.6 kV/cm; 5 µs × 100, by 1.8 ± 0.1 Log_10_ CFU/ml and 2.6 ± 0.1 Log_10_ CFU/ml, respectively. In addition, they noted that treatment of the same culture twice with the PEF caused a decrease in the survival rate of the bacteria. The authors of the present publication also observed a decrease in the total number of microorganisms in the culture subjected to multiple PEF treatment (by 8 × 10^12^ CFU/ml), compared to the cultures treated with PEF after 20 h of culturing (Table 2). That decrease, however, was not statistically significant. Tang *et al*. [38], in a study on fermented soymilk enriched with calcium (120 mg/100 ml), after 24 h achieved a survival rate above 8.8 log_10_ CFU/ml for all analyzed strains. Huang *et al*. [39] observed that an addition of CaCl_2_ increases the survival rate of lactic acid bacteria as a result of heating. Vaessen *et al*. [15] noted a decrease in the survival rate of *L. plantarum* WCFS1 by 50%at field strength of 10 and 12.5kV/cm. By contrast, field strength of 7.5 kV/cm caused a high survival rate of bacteria (62%-93%) and considerable accumulation of trehalose in cells. Inactivation of 2 units of Log_10_ *Escherichia coli* ATCC 35218 in orange juice was obtained only at 22 kV/cm i 45°C [40]. Field strength of 34.8 kV/cm caused a reduction of *L. plantarum* in beer by 4-Log_10_ [41]. The authors of this publication did not conduct any analyses at such a high field strength. In a study by Góral *et al*. [23] the total average number of bacteria at the particular stages of the study was lower than that obtained in the present study. The highest viability of *L. rhamnosus* B 442 (1.13·10^11^) was achieved at the lowest concentration of zinc ions applying a PEF. When utilizing a pulsed electric field with optimized parameters, the total number of microorganisms was strongly varied. Also, such a high field strength and pulse width were not applied.

Pankiewicz *et al*. [42] observed similar relationships with the application on calcium orange to induce fluorescence of cells of *S. cerevisiae* supplemented with calcium ions. Those authors observed that, as in the case of the study presented in this publication, fluorescence of the control sample was several-fold lower than that of the sample enriched in Ca^2+^ with the use of a PEF. In microscopy specimens of yeast, the distribution of calcium ions in a cell could be observed, due to their larger size compared to bacteria.

Optimization of calcium concentration in the medium and of the parameters of the pulsed electric field allowed achievement of over 300% higher concentration of Ca^2+^ ions in cells of *L.rhamnosus* B 442 as compared to the control culture K2. The highest accumulation (7.30 mg/g d.m.) was achieved at the concentration of 200 µg/ml of medium and at the following PEF parameters: field strength of 3.0 kV/cm, exposure time of 10 min and pulse width of 75 µs. Optimization of the time after which the cultures were treated with a pulsed electric field did not cause significant changes in the accumulation of Ca^2+^ ions. This shows that the culturing time in the first stage of the study was correctly chosen. Biomass in cultures subjected to electroporation and supplemented with calcium ions varied from 0.09 g/g d.m. to 0.252 g/g d.m. Its lowest value was noted in the culture subjected to multiple PEF treatments due to the long breaks in culturing and to the stress caused by five-time electroporation. The highest value was obtained at pulse width of 75 µs, at which the highest accumulation of calcium ions was achieved. Mean content of biomass in cells enriched with calcium ions but not electroporated was higher by 0.02 g/g d.m. compared to cultures subjected to PEF treatment. The total number of microorganisms was high (considerably higher than the minimum recommended for probiotic food) and varied from 10^10^ to 10^12^ CFU/ml. The acquired confocal microscopy images provide evidence that calcium ions were located within bacterial cells, and electroporation contributed to an increase in the effectiveness of the process of bioaccumulation of calcium ions.

## Figures and Tables

**Table 1:** Effect on Ca^2+^ concentration in medium of total number of *L. rhamnosus* B 442 with and without PEF treatment.

Concentration of calcium [μg/mL]	Total microorganisms [CFU/mL]		

	Without PEF	With PEF	
K1	5.62·10^11^^a^	-
10	1.78·10^11^^a^	2.89·10^11^^a^
25	4.92·10^10^^a^	1.51·10^12^^b^
100	5.21·10^10^^a^	3.13·10^11^^a^
150	1.75·10^11^^a^	1.55·10^12^^b^
200	1.75·10^12^^b^	4.82·10^10^^a^
400	5.88·10^10^^a^	8.47·10^11^^b^
500	1.55·10^10^^a^	3.09·10^10^^a^
750	5.96·10^11^^ab^	4.45·10^10^^a^
1000	6.08·10^10^^a^	2.46·10^11^^a^

^a,b^Means in the same column indicated by different letters were significantly different (*p* value < 0.05).

**Table 2:** Effect of electric field strength, time of exposure to PEF, pulse width and time after cultures were treated with PEF on total number of microorganisms of *L. rhamnosus* B 442.

Total microorganisms [CFU/mL]									

Low electric field strength [kV/cm]		High electric field strength [kV/cm]		Time of electroporation [min]		Pulse width [μs]		Time after cultures were treated PEF [hours h]	
K1	6.60•10^11^^a^	K1	6.56•10^11^^a^	K1	9.55•10^10^^a^	K1	6.47•10^11^^a^	K1	9.65•10^11^^c^
K2	6.47•10^11^^a^	K2	6.50•10^11^^a^	K2	1.06•10^10^^a^	K2	6.07•10^11^^a^	K2	2.41•10^12^^d^
0.1	1.19•10^10^^a^	1.5	1.90•10^11^^a^	5	3.11•10^10^^a^	10	5.84•10^11^^a^	8	2.13•10^10^^a^
0.2	9.40•10^11^^a^	2.0	2.12•10^12^^a^	10	9.09•10^9^^a^	20	4.44•10^11^^a^	12	1.45•10^10^^a^
0.3	2.99•10^10^^a^	3.0	2.97•10^10^^a^	15	5.82•10^10^^a^	50	1.05•10^11^^a^	16	3.27•10^10^^a^
0.4	4.82•10^11^^a^	4.0	9.76•10^11^^a^	20	1.92•10^10^^a^	75	4.42•10^11^^a^	20	1.00•10^11^^a^
0.5	3.25•10^11^^a^	5.0	1.61•10^12^^a^	25	1.15•10^12^^a^	100	4.78•10^11^^a^	24	3.64•10^11^^b^
1.0	3.15•10^11^^a^	6.0	1.55•10^12^^a^			125	6.62•10^12^^b^	multiple	2.00•10^10^^a^
						150	5.44•10^12^^b^		

Control cultures: K1 - without calcium and without PEF treatment; K2 - with calcium (200 μg/ml) and without PEF treatment.

^a,b^Means in the same column indicated by different letters were significantly different (*p* value < 0.05).

## References

[1] Vavrusova M, Skibsted LH (2014). Calcium nutrition. Bioavailability and fortification. LWT - Food Sci. Technol..

[2] Vavrusova M, Danielsen BP, Garcia AC, Skibsted LH (2018). Codissolution of calcium hydrogenphosphate and sodium hydrogencitrate in water. Spontaneous supersaturation of calcium citrate increasing calcium bioavailability. J. Food Drug Anal..

[3] Titchenal CA, Dobbs J (2007). A system to assess the quality of food sources of calcium. J. Food Compost Anal..

[4] Camara-Martos F, Amaro-Lopez MA (2002). Influence of dietary factors on calcium bioavailability. Biol. Trace Elem. Res..

[5] Bolland MJ, Grey A, Avenell A, Gamble GD, Reid IR (2011). Calcium supplements with or without vitamin D and risk of cardiovascular events: reanalysis of the Women's Health Initiative limited access dataset and meta-analysis. BMJ.

[6] Reid IR, Bristow SM (2016). Calcium fortified foods or supplements for older people?. Maturitas.

[7] Rooney MR, Michos ED, Hootman KC, Harnack L, Lutsey PL (2018). Trends in calcium supplementation, National Health and Nutrition Examination Survey (NHANES) 1999-2014. Bone.

[8] Cha JY, Cho YS (2009). Determination of optimal conditions for zinc hyperaccumulation by *Saccharomyces cerevisiae* FF-10. J. Korean Soc. Appl. Biol. Chem..

[9] Toepfl S, Siemer C, Saldaña-Navarro G, Heinz V (2014). Overview of pulsed electric fields processing for food. Emerging technologies for food processing.

[10] Wang MS, Wang LH, Bekhit AEDA, Yang J, Hou ZP, Wang YZ (2018). A review of sublethal effects of pulsed electric field on cells in food processing. J. Food Eng..

[11] Suchanek M, Olejniczak Z (2018). Low field MRI study of the potato cell membrane electroporation by pulsed electric field.? J. Food Eng..

[12] Escoffre JM, Portet T, Wasungu L, Teissié J, Dean D, Rols MP (2009). What is (still not) known of the mechanism by which electroporation mediates gene transfer and expression in cells and tissues. Mol. Biotechnol..

[13] Dermol-Černe J, Miklavčič D, Reberšek M, Mekuč P, Bardet SM, Burke R (2018). Plasma membrane depolarization and permeabilization due to electric pulses in cell lines of different excitability. Bioelectrochemistry.

[14] Teissie J, Golzio M, Rols MP (2005). Mechanisms of cell membrane electropermeabilization: a minireview of our present (lack of) knowledge. Biochim. Biophys. Acta.

[15] Vaessen EMJ, den Besten HMW, Patra T, van Mossevelde NTM, Boom RM, Schutyser MAI (2018). Pulsed electric field for increasing intracellular trehalose content in *Lactobacillus plantarum* WCFS1. Innov. Food Sci. Emerg. Technol..

[16] Kolosnjaj-Tabi J, Gibot L, Fourquaux I, Golzio M, Rols MP (2018). Electric field-responsive nanoparticles and electric fields: physical, chemical, biological mechanisms and therapeutic prospects. Adv. Drug Deliv. Rev..

[17] Phez E, Gibot L, Rols MP (2016). How transient alterations of organelles in mammalian cells submitted to electric field may explain some aspects of gene electrotransfer process. Bioelectrochemistry.

[18] Hristov K, Mangalanathan U, Casciola M, Pakhomova ON, Pakhomov AG (2018). Expression of voltage-gated calcium channels augments cell susceptibility to membrane disruption by nanosecond pulsed electric field. Biochim. Biophys. Acta Biomembr..

[19] Liu ZW, Zeng XA, Sun DW, Han Z (2014). Effects of pulsed electric fields on the permeabilization of calcein-filled soybean lecithin vesicles. J. Food Eng..

[20] Jorhem L, Engman J (2000). Determination of lead, cadmium, zinc, copper, and iron in foods by atomic absorption spectrometry after microwave digestion: NMKL1 collaborative study. J. AOAC Int..

[21] American Public Health Association (1993). Standard Methods for the Examination of Dairy Products.

[22] Góral M, Pankiewicz U (2017). Effect of pulsed electric fields (PEF) on Accumulation of magnesium in *Lactobacillus rhamnosus* B 442 Cells. J. Membr. Biol..

[23] Góral M, Pankiewicz U, Sujka M, Kowalski R (2019). Bioaccumulation of zinc ions in *Lactobacillus rhamnosus* B 442 cells under treatment of the culture with pulsed electric field. Eur. Food Res. Technol..

[24] Marafon AP, Sumi A, Alcântara MR, Tamime AY, De Oliveira MN (2011). Optimization of the rheological properties of probiotic yoghurts supplemented with milk proteins. LWT - Food Sci. Technol..

[25] Raso J, Frey W, Ferrari G, Pataro G, Knorr D, Teissie J (2016). Recommendations guidelines on the key information to be reported in studies of application of PEF technology in food and biotechnological processes. Innov. Food Sci. Emerg. Technol..

[26] Silve A, Leray I, Poignard C, Mir LM (2016). Impact of external medium conductivity on cell membrane electro-permeabilization by microsecond and nanosecond electric pulses. Sci. Rep..

[27] Barba FJ, Parniakov O, Pereira SA, Wiktor A, Grimi N, Boussetta N (2015). Current applications and new opportunities for the use of pulsed electric fields in food science and industry. Food Res. Int..

[28] Pankiewicz U, Jamroz J (2013). Application of pulsed electric field for enrichment of *Saccharomyces cerevisiae* cells with calcium ions. Ital. J. Food Sci..

[29] Roman J, Gniewosz M, Mantorska J (2009). Comparison of magnesium binding, growth and acidifying properties of *Lactobacillus brevis* and *Lactobacillus plantarum* in an environment with elevated magnesium concentration. Acta Scient. Pol. Biotechnol..

[30] Mörschbächer AP, Dullius A, Dullius CH, Bandt CR, Kuhn D, Brietzke DT (2018). Assessment of selenium bioaccumulation in lactic acid bacteria. J. Dairy Sci..

[31] Mrvčić J, Prebeg T, Barišić L, Stanzer D, Bačun-Družina V, Stehlik-Tomas V (2009). Zinc binding by lactic acid bacteria. Food Technol. Biotechnol..

[32] Mrvcic J, Stanzer D, Bacun-Druzina V, Stehlik-Tomas V (2009). Copper binding by lactic acid bacteria (LAB). Biosci. Microflora..

[33] Sánchez B, Delgado S, Blanco-Míguez A, Lourenço A, Gueimonde M, Margolles A (2017). Probiotics, gut microbiota, and their influence on host health and disease. Mol. Nutr. Food Res..

[34] Ouwehand AC, Salminen S, Isolauri E, Siezen RJ, Kok J, Abee T, Schaafsma G (2002). Probiotics: an overview of beneficial effects. Lactic Acid Bacteria: Genetics, Metabolism and Applications.

[35] Ziarno M, Wieclawski S (2006). The effect of an addition of calcium lactate on the growth of lactic acid fermentation bacteria in MRS broth and in milk. Żywność Nauka Technologia Jakość.

[36] Pirkul T, Temiz A, Erdem YK (1997). Fortification of yoghurt with calcium salts and its effect on starter microorganisms and yoghurt quality. Int. Dairy J..

[37] Seratlić S, Bugarski B, Nedović V, Radulović Z, Wadsö L, Dejmek P, Galindo FG (2013). Behavior of the surviving population of *Lactobacillus plantarum* 564 upon the application of pulsed electric fields. Innov. Food Sci. Emerg. Technol..

[38] Tang AL, Shah NP, Wilcox G, Walker KZ, Stojanovska L (2007). Fermentation of calcium-fortified soymilk with *Lactobacillus*: effects on calcium solubility, isoflavone conversion, and production of organic acids. J. Food Sci..

[39] Huang S, Yang Y, Fu N, Qin Q, Zhang L, Chen XD (2014). Calcium-aggregated milk: a potential new option for improving the viability of lactic acid bacteria under heat stress. Food Bioproc. Tech..

[40] Gurtler JB, Rivera RB, Zhang HQ, Geveke DJ (2010). Selection of surrogate bacteria in place of *E. coli* O157: H7 and *Salmonella* Typhimurium for pulsed electric field treatment of orange juice. Int. J. Food Microbiol..

[41] Ulmer HM, Heinz V, Gänzle MG, Knorr D, Vogel RF (2002). Effects of pulsed electric fields on inactivation and metabolic activity of *Lactobacillus plantarum* in model beer. J. Appl. Microbiol..

[42] Pankiewicz U, Jamroz J, Sujka M, Kowalski R (2015). Visualization of calcium and zinc ions in *Saccharomyces cerevisiae* cells treated with PEFs (pulse electric fields) by laser confocal microscopy. Food Chem..

